# Takotsubo Cardiomyopathy: A COVID-19 Complication

**DOI:** 10.7759/cureus.22803

**Published:** 2022-03-03

**Authors:** Pejmahn Eftekharzadeh, Ankit Patel, Elena Sokolova, Aaron Rodas, Shahzad Ahmed

**Affiliations:** 1 Internal Medicine, Lower Bucks Hospital, Bristol, USA; 2 Cardiology, Lower Bucks Hospital, Bristol, USA

**Keywords:** takotsubo cardiomyopathy, spike s1 protein, spike protein, ace inhibitors and covid-19, acute coronary syndrome, heart failure, covid 19, stress induced cardiomyopathy

## Abstract

COVID-19 started as an unknown viral illness and has been a challenging pandemic to overcome. The virus has been associated with multiple organ involvement, including the heart. Takotsubo cardiomyopathy (TSCM), a stress cardiomyopathy, is an uncommon complication in patients diagnosed with COVID-19. The pathogenesis is historically a result of stress onto the body that leads to a catecholamine surge. However, COVID-19 may cause direct damage to the cardiac myocytes via spike protein and angiotensin-converting enzyme 2 (ACE2) receptors which can further exacerbate the stressful insult on the patient and lower the threshold for developing TSCM. In this case report, we discuss a 94-year-old female who presented with signs and symptoms of acute coronary syndrome but, upon cardiac catheterization, was found to have basal hypercontraction with apical ballooning, consistent with TSCM.

## Introduction

In 2019, the severe acute respiratory syndrome coronavirus 2 (SARS-CoV-2) emerged in Asia and, due to its easy transmissibility, has spread globally causing one of the worst pandemics in history [1]. The virus has been associated with numerous pulmonary complications such as pneumonia and acute respiratory distress syndrome and often leads to respiratory failure. While the virus historically affects the pulmonary system, it can affect various other organs including the heart. As cardiac manifestations evolve, we learn that COVID-19 can induce arrhythmias, myocardial injury, heart failure, and acute coronary syndrome [2]. One relatively uncommon manifestation is stress cardiomyopathy, Takotsubo cardiomyopathy (TSCM), which has been reported with increasing frequency [3]. TSCM is more prevalent in females and is often triggered by physical and/or emotional stress on a patient. Some examples of physical stress include infection, fracture, and/or acute respiratory distress, while examples of emotional stress include panic, anxiety, grief, interpersonal conflict, and/or anger [4]. Manifestations for TSCM include transient left ventricular wall motion abnormalities without evidence of coronary artery disease and/or plaque rupture, presence of EKG changes, as well as an elevated troponin level [4]. In this article, we discuss a case of a female patient who developed TSCM as a result of contracting COVID-19.

In a literature review using PubMed, TSCM is not uncommon in the setting of COVID-19. In a review article, the author discusses several case reports as well as retrospective studies in patients with stress cardiomyopathy. The incidence of TSCM in the general population was between 1.5% and 1.8% before the beginning of the pandemic. However, during the pandemic, this number rose to 7.75%. Common comorbidities of these patients in the article showed that two-thirds of them did have hypertension and almost half of them had a history of diabetes mellitus. Only one case report was noted to have a history of anxiety disorder. There was a female predominance of 73% among the cases [5]. In another systemic review, the author describes 40 articles with a total of 52 cases of TSCM who were positive for COVID-19. In this review, there was also a female predominance of 59.6% [6]. However, in one last review of 118 patients with COVID-19, all five cases of TSCM were men [7]. 

## Case presentation

**Figure 1:**
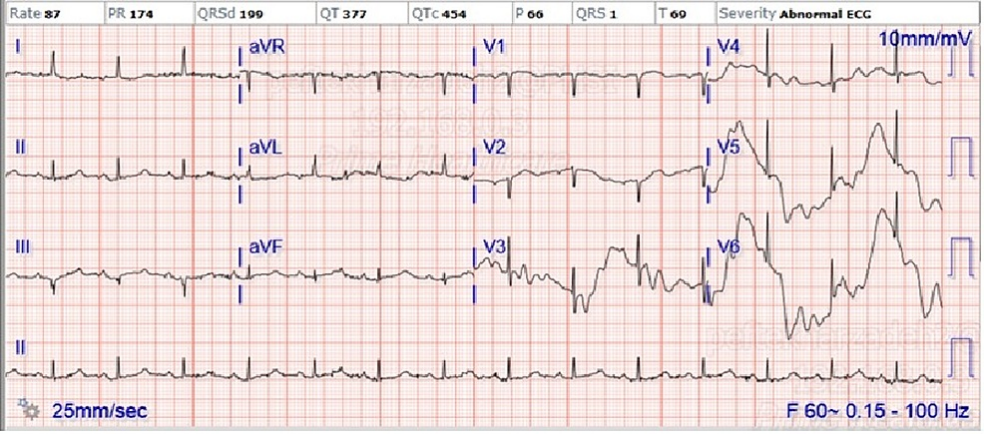
Initial EKG on presentation shows normal sinus rhythm without ST changes QTc: 454. aVR: augmented vector right, aVL: augmented vector left, aVF: augmented vector foot.

A 94-year-old white female with past medical history of anxiety disorder is brought to the emergency room (ER) with complaints of shortness of breath. She tested positive for COVID-19 one week prior at a local urgent care facility after she had a confirmed exposure and developed nonspecific symptoms of fatigue and decreased appetite. She was instructed to self-quarantine, monitor for worsening symptoms, and comply with supportive therapy. Eventually, she developed respiratory distress which worsened for one week, leading her to contact the emergency medical service (EMS). Upon EMS arrival to the patient’s home, she was observed in severe respiratory distress with an oxygen saturation of 70% on room air and was placed on a nonrebreather (NRB) mask and transported to the hospital. On arrival, her observed values were as follows: blood pressure of 196/93 mmHg, pulse of 118 beats per minute (bpm), rectal temperature of 99.8°F, respirations of 46 breaths per minute, and pulse oxygen of 96% on the NRB mask. She was initially encephalopathic and not oriented to person, place, or time. The cardiovascular exam showed tachycardia without jugular venous distension or lower extremity edema. Pulmonary/chest exam showed accessory muscle use with rales and rhonchi. Given these rales and rhonchi, the patient was trialed on noninvasive positive pressure ventilation (NIPPV) while in the ER which improved her respiratory distress and her mental status. In the ER, a chest x-ray showed clear lungs with cardiomegaly. EKG on presentation is shown in Figure 1.

Troponin on presentation was 0.05 ng/L (reference: 0.000-0.060 ng/L), peaked at 0.188 ng/L, and then subsequently downtrended. Her N-terminal-prohormone brain natriuretic peptide (NT-proBNP) was 1,874 pg/ml. She had no documented diagnosis of heart failure. Her blood pressure was markedly elevated on admission and was successfully treated with IV labetalol as needed. She was initially started on antibiotics, ceftriaxone and azithromycin, for possible superimposed community-acquired pneumonia but later discontinued after her polymerase chain reaction (PCR) for COVID-19 was positive. She was also subsequently started on empiric therapy with remdesivir and steroids.

**Figure 2:**
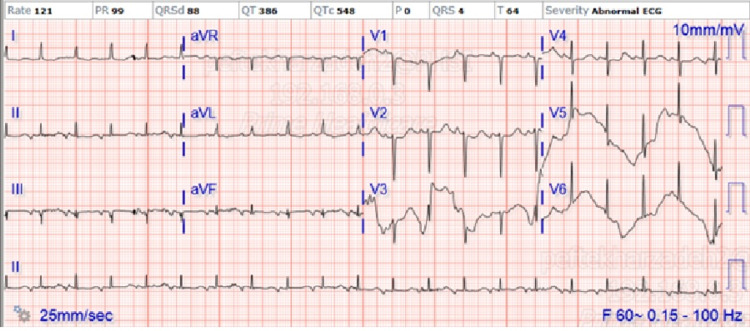
EKG showing sinus tachycardia without any acute ST changes aVR: augmented vector right, aVL: augmented vector left, aVF: augmented vector foot.

Throughout day 1, trials of oxygen de-escalation were attempted without success and NIPPV was continued. During the evening of day 1, the patient became intermittently agitated by waving her arms, making nonsensical verbiage, climbing out of bed, and tampering with NIPPV causing her to become hypoxic. A trial of redirection was successful. Occasionally, her intermittent restlessness and agitation induced tachycardia, and another EKG was done as shown in Figure 2.

**Figure 3:**
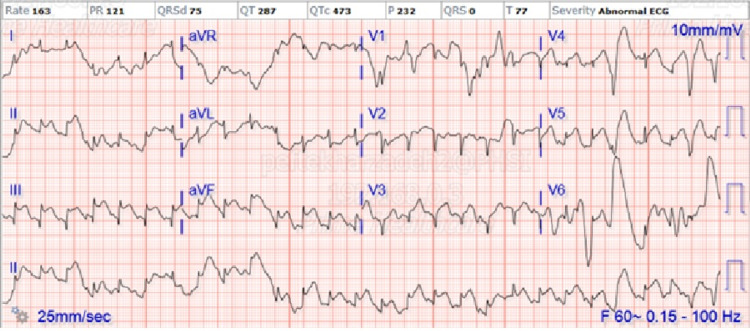
EKG showing rate of 163 with ST segment elevation in inferior lateral leads II, III, aVF, and V5 aVR: augmented vector right, aVL: augmented vector left, aVF: augmented vector foot.

In the evening of hospital day 2, the patient became acutely more anxious and restless. The telemetry monitor showed a sinus tachycardia of 160 bpm, a systolic blood pressure of 160 mmHg, and a respiratory rate of 36 breaths per minute despite NIPPV therapy. Immediate EKG showed ST elevation in inferior lateral leads II, III, augmented vector foot (aVF), and V5 as illustrated in Figure 3.

**Figure 4:**
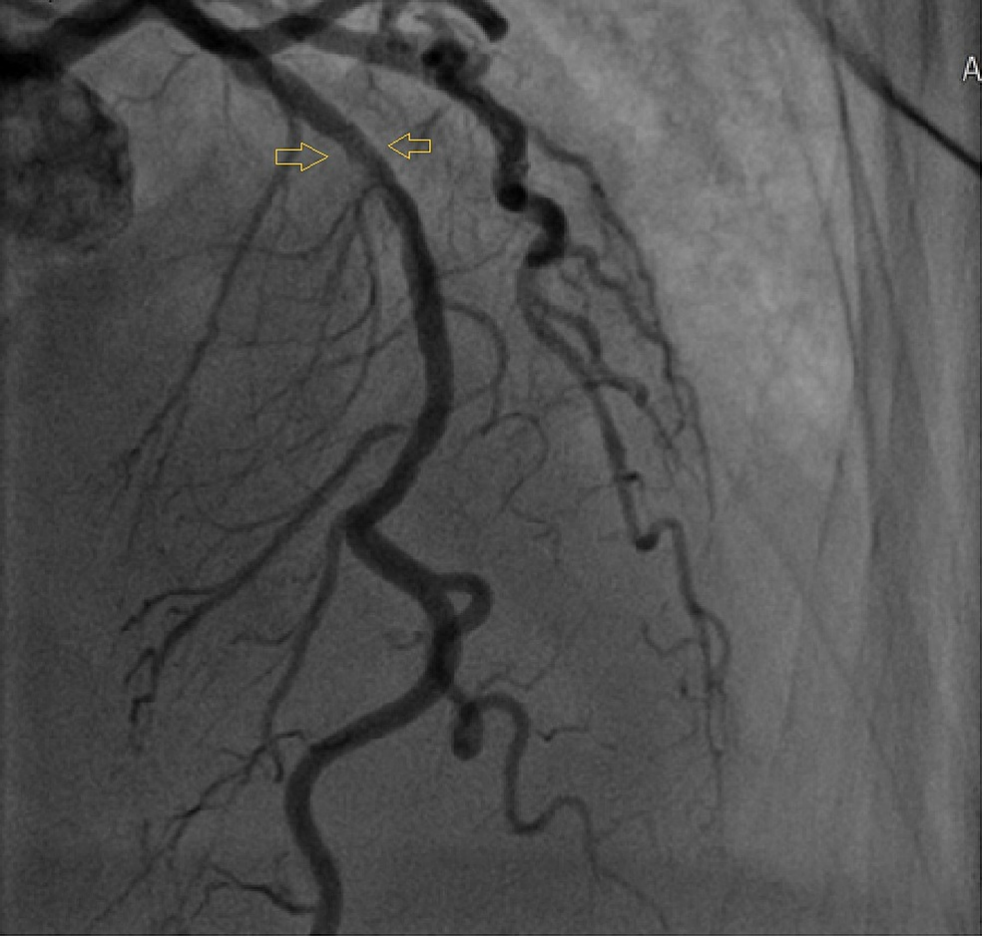
Left cardiac catheterization showing an approximately 40% proximal to mid-LAD lesion Yellow arrows show mid-LAD lesion. LAD: left anterior descending.

An ST segment elevation myocardial infarction (STEMI) alert was triggered, and immediate labs revealed a troponin of 4.237 ng/L. Patient was given aspirin 300 mg rectally and immediately taken to the cardiac catheterization lab. Emergent left heart catheterization was done to assess coronary artery patency which showed only a 40% proximal to mid-left anterior descending (LAD) lesion without any severe obstruction as noted in Figure 4.

**Figure 5:**
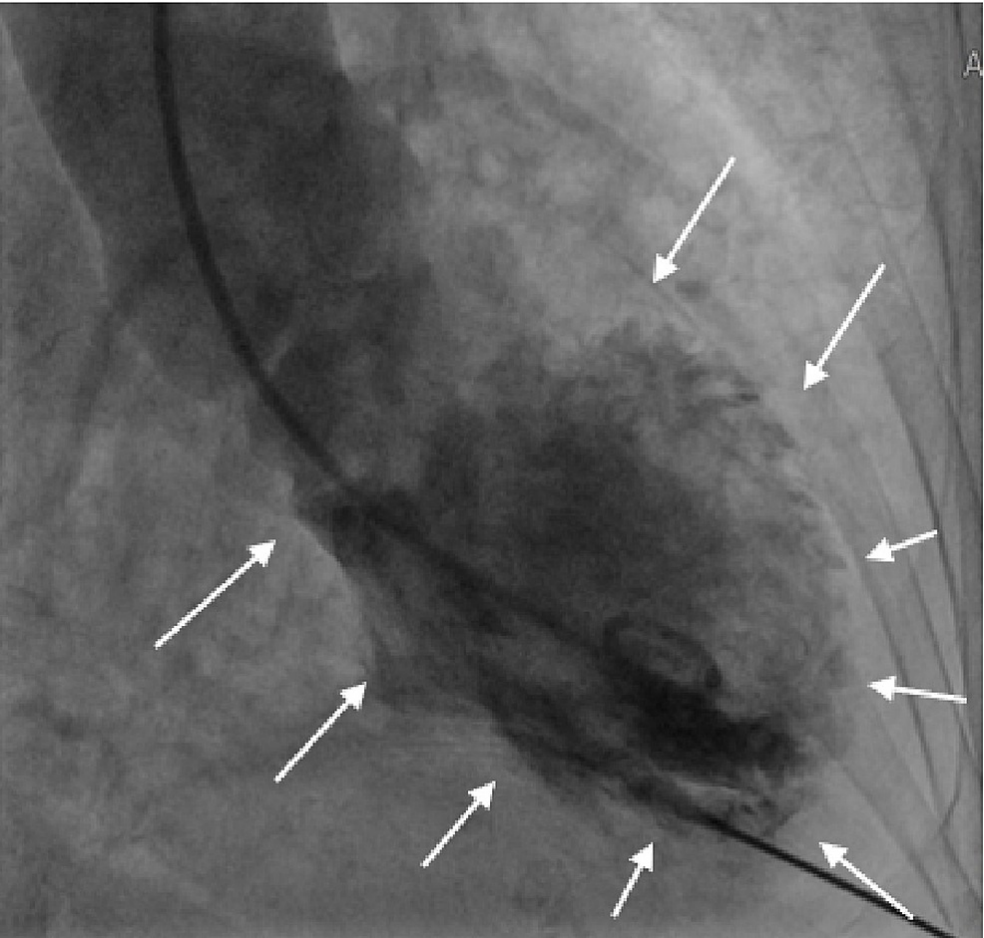
Cardiac catheterization during diastole showing myocardial tissue relaxation White arrows show left ventricle in diastole.

**Figure 6:**
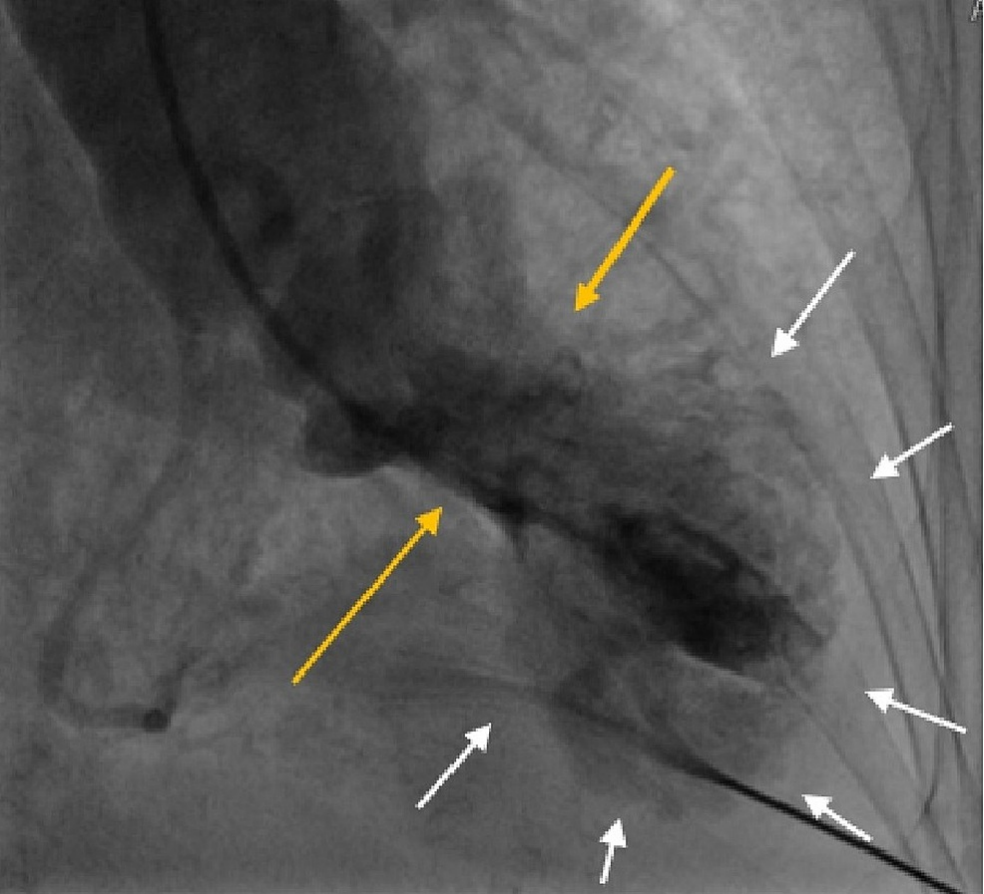
Cardiac catheterization during systole with basal hypercontraction and apical left ventricular (LV) ballooning out Gold arrows show basal hypercontraction. White arrows show apical ballooning.

Upon further investigation, moderate left ventricular (LV) dysfunction with apical ballooning was noted during the left heart chamber assessment as depicted below. Note, Figures 5, 6 represent cardiac diastole and systole, respectively.

**Figure 7:**
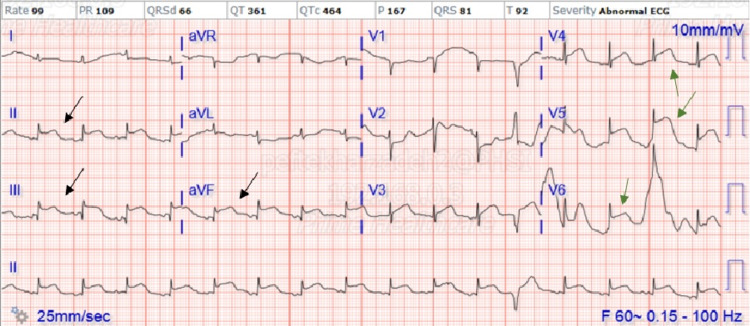
EKG status postcardiac catheterization showing persistent ST elevations in inferior and lateral lead distribution Black arrows show ST elevations in inferior leads. Green arrows show ST elevations in lateral leads. aVR: augmented vector right, aVL: augmented vector left, aVF: augmented vector foot.

No further interventions were done, and the patient returned to the intensive care unit (ICU). Repeat EKG is shown in Figure 7 depicting persistent ST elevations in an inferolateral lead distribution.

Given the patient's clinical course, the present on admission (POA), with collaborative discussions with family, wished the patient to be made do not resuscitate (DNR) and do not intubate (DNI). They also requested inpatient hospice care, and the patient ultimately passed away the following day.

## Discussion

TSCM is a syndrome in which the myocardium undergoes a transient change, with the LV taking an apical ballooning shape, and is associated with a reduced ejection fraction, elevated cardiac enzyme levels, and signs of ischemia on EKG in a nonspecific vascular distribution. Risk factors for TSCM include postmenopausal women and any emotional and/or physical stress [8]. The clinical manifestation of TSCM is not only presented with acute substernal chest pain but also can have associated dyspnea or syncope. Some patients develop signs of heart failure, tachyarrhythmias, sudden cardiac arrest, or significant mitral regurgitation, while as many as 10% develop cardiogenic shock and respiratory distress [9]. The myopathy adopts its name “Takotsubo” coming from the Japanese word “octopus” owing to the heart's image during cardiac catheterization for taking the shape of an octopus trap when contrast is injected into the LV to show apical ballooning [10].

Our 94-year-old patient is in the target population that predisposes her to TSCM given her age and gender. First, COVID-19 does impose a physical stress on the body, namely acute respiratory failure due to ongoing hypoxia as the pathogenesis of the virus progresses. Furthermore, there are emotional stressors that exacerbate symptoms, as witnessed by the patient. The first is wearing an NIPPV machine, in which the tight fit can impose fear or panic due to claustrophobia. The patient also has a history of anxiety disorder which predisposes her to some baseline emotional stress. Lastly, the knowledge of being infected with COVID-19, which has significant mortality, can induce anxiety and stress in itself. Thus, it may be concluded that contracting COVID-19 can couple the physical and emotional stressors to synergistically lower the threshold to develop TSCM.

**Figure 8:**
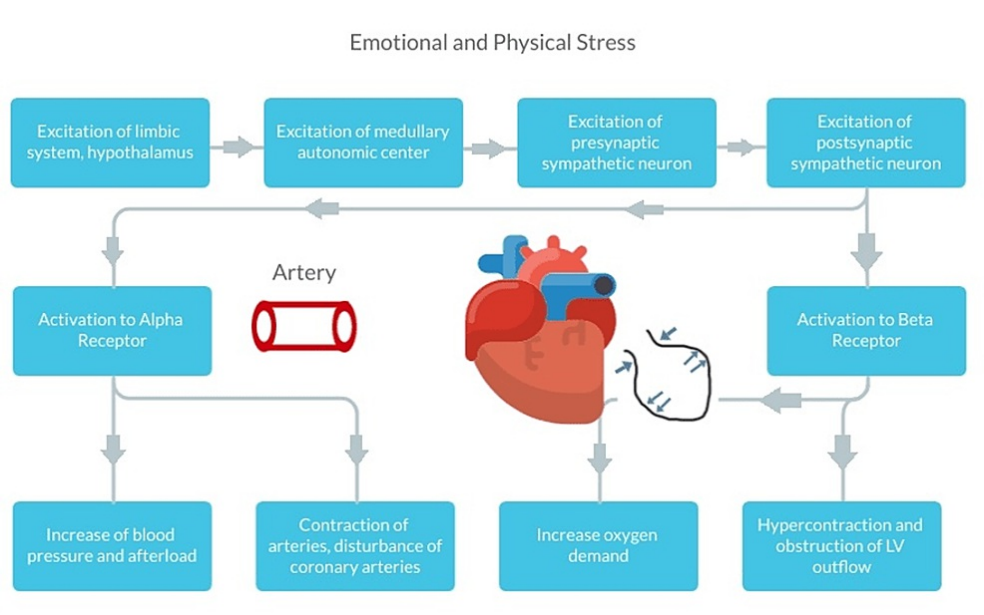
Pathogenesis of TSCM α-receptors cause contraction of coronary arteries inducing ischemia on cardiac membrane, while β1- and β2-receptors cause an increase in oxygen demand and increase in mechanical wall stress. Gray/purple arrows indicate cardiac chamber morphological changes during TSCM. TSCM: Takotsubo cardiomyopathy, LV: left ventricle.

The current pathophysiology of TSCM is poorly understood. Figure 8 displays an indirect pathway showing a catecholamine surge from the emotional and/or physical stress. The response begins in the hypothalamus and triggers sympathetic norepinephrine and epinephrine release. These catecholamines work on α-receptors to contract coronary arteries inducing ischemia on cardiac membrane. They also work on β1- and β2-receptors to alter myocardial contractility, increase oxygen demand, and increase mechanical wall stress.

**Figure 9:**
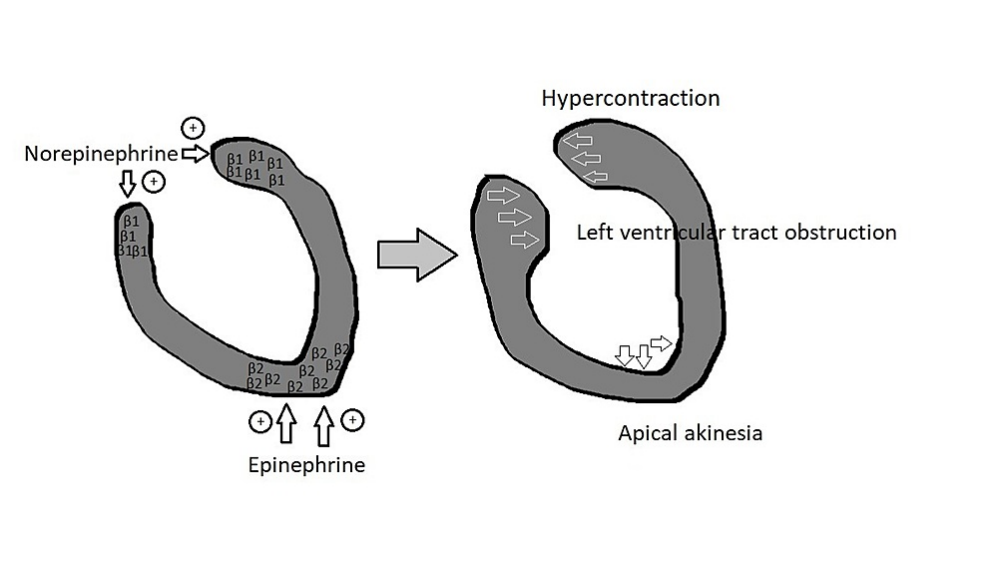
More β1-receptors on the left ventricular base that are innervated by norepinephrine to induce hypercontraction by triggering the G-protein stimulatory pathway More β2-receptors on the cardiac apex are innervated by epinephrine that triggers the G-protein inhibitory pathway, producing apical akinesia. β1: beta-1 receptors that undergo G-protein stimulatory intervention on basal left ventricle when innervated by norepinephrine. β2: beta-2 receptors that undergo G-protein inhibitory intervention on apical left ventricle when innervated by epinephrine. White outlined arrows indicate basal hypercontraction. Black outlined arrows indicate apical akinesia.

Figure 9 explains the characteristic of the “octopus trap” heart. Norepinephrine triggers the β1-receptors on the LV base which in turn acts on a G-protein stimulatory pathway to induce hypercontractions, while epinephrine works on β2-receptors on the cardiac apex by G-protein inhibitory pathway, stimulating hypocontraction [11]. Unfortunately, catecholamine levels were not obtained in this patient given the family's hospice request after the cardiac catheterization.

Next, we discuss a more direct method of physical stress on the heart through a unique protein receptor. SARS-CoV-2 uses a spike S1 protein to enable the attachment of the virion to the cell membrane of the host by interacting with host angiotensin-converting enzyme 2 (ACE2) receptors [12]. ACE2 receptors are highly expressed on mammalian cardiovascular tissue including endothelial cells, cardiac myocytes, fibroblasts, and smooth muscle cells [13-15]. Once the virus binds to the ACE2 receptors of smooth muscle cells on coronary arteries using this spike S1 protein, this can negatively downregulate the receptor's function causing an accumulation of angiotensin II to cause vasoconstriction, oxidative stress, and inflammation [12]. The vasoconstriction worsens the coronary ischemia that is evident in TSCM. Thus, the accumulation of angiotensin II not only increases the catecholamine surge, as mentioned above, and induces the coronary ischemia but can also promote transient vasoconstriction and worsen the ischemia. As a result, cardiac enzymes will become elevated, a hallmark finding in TSCM. All the above processes pose a direct physical myocardial stress onto the heart, which can lower the threshold for inducing TSCM.

## Conclusions

COVID-19 can predispose patients to develop TSCM as the virus plays numerous roles in physical and emotional stress. The pathophysiology can be via a catecholamine surge which can cause an increase in blood pressure, increase in coronary artery contractility, and increase in oxygen demand leading to mechanical stress. However, through the virus’s spike S1 protein, it can enter cardiac myocytes and coronary vessels by binding to angiotensin-converting enzyme 2 receptors on the heart. Once it enters the cells, the virus can disrupt normal physiologic pathways and cause additional cardiac stress via vasoconstriction, oxidative stress, and inflammation. Both stressors can work simultaneously and synergistically to lower the threshold to develop TSCM in COVID-19 patients.
